# Incorporation of prior knowledge and habits while solving anagrams

**DOI:** 10.16910/jemr.15.5.5

**Published:** 2022-11-05

**Authors:** Jesse Murray, Andrew Sutter, Angelia Lobifaro, Graham Cousens, Minjoon Kouh

**Affiliations:** Drew University

**Keywords:** Eye movement, eye tracking, anagram, puzzle, prior knowledge, language statistics, bigram, n-gram

## Abstract

Games and puzzles provide a valuable context for examining human problemsolving behavior. We recorded and analyzed the sequence of letters viewed by the participants of our study while they were solving anagram puzzles. The goal was to examine and understand how people's linguistic habits and prior knowledge influenced their eye movements. The main findings of this study are: (1) People's stereotypical habit of scanning (e.g., adjacent or top viewing) strongly influences their solution-seeking behavior. (2) People tend to incorporate their prior knowledge of letter statistics in a reasonable way, such as looking less frequently at letter combinations that are uncommon in the English language.

## Introduction

How do humans perform complex tasks and solve problems? How do we incorporate insights from prior knowledge and experiences? How do we adapt and employ other problem-solving approaches to a new problem? These are fascinating questions for the studies of not only human and animal cognition, but also artificial intelligence, and they are of interest to the booming industry of game development. Furthermore, it is important to be aware of our own problem-solving approaches, which may be limited and may not always be optimal. Such awareness would allow us to consider expansively other problem-solving strategies, as we address many dire challenges in the world.

Games and puzzles provide a useful context for answering those questions, since they are highly engaging and complex yet simple enough to generate a wide range of behaviors, which can be observed, quantified, and controlled. Experimental psychology has a productive history of using games in numerous studies (8; 9; 11; 15). It is also worth noting that many recent breakthroughs in the field of artificial intelligence were made in the context of human games such as chess, go, Jeopardy!, and video games (10; 23; 26).

Observing the eye movement of participants as they attempt to solve a problem or perform a complex task has a long history (33; 19; 25). The pioneering studies by Yarbus (33) involved participants examining a painting and inferring the material circumstances, ages, and so forth from the scene. Other recent eye movement research has looked at a variety of participants performing laparoscopic surgery, solving science ordering problems, etc., just to name a few (22; 28; 17; 20).

Eye movement data is particularly interesting and rich with implications about underlying cognitive processes. Since what was viewed from the current gaze guides the brain to perform the next eye movement, it forms an open, dynamic cycle of information gathering and processing. The temporal trajectory of saccades and the resulting history of fixations give the researchers a glimpse into the problem-solving strategies of participants.

In this study, participants solved a series of anagram puzzles, each of which consisted of a set of randomly placed letters that make up a word. Such a word game is classic, engaging, and popular. A modern remake of a word-guessing game has recently gone viral (29). One advantage of anagrams is that the search space of their solutions is well-defined because each solution word must use each of the given letters precisely once, and all letters have to be used, although it is also possible and interesting to introduce a distractor letter to avoid using, as done in other studies (8; 9).

The present study sought to utilize this approach to explore the impact of habitual scanning patterns and prior knowledge on problem-solving. Previous findings demonstrating positional gaze biases in other paradigms (e.g., 7) suggest that anagram solution strategies might be influenced by habitual scanning patterns or by gaze biases. We also sought to build on previous research showing that linguistic knowledge influences problem-solving performance and visual scanning patterns in anagram tasks (9; 21) by determining whether implicit knowledge of letter sequence probability would influence eye movements during task performance.

We predicted that (1) participants’ stereotypical habit of left-to-right scanning during the reading would strongly influence their solution-seeking behavior, and (2) participants would tend to incorporate their prior knowledge of letter statistics by looking more frequently at letter combinations that are common in the English language, and (3) the gaze patterns for the letter sequences suggestive of solutions would be different on average between correctly-solved trials compared to unsolved trials.

## Methods

### Participants

A convenience sample of college students was recruited by word of mouth. Data are reported here for 29 participants who were proficient in the English language. Procedures were conducted in accordance with The Declaration of Helsinki and were approved by the Drew University Institutional Review Board.

### Apparatus

Images of anagram puzzles were presented on Tobii T60 Eye Tracker (Tobii Pro), a 17-inch, 60 Hz monitor with a resolution of 1280 by 1024 pixels. Individual letters subtended approximately a half degree and the entire anagram approximately 30 degrees. The participant's eyes were positioned approximately 60 cm from the screen (at about an arm's length). Tobii T60 uses an infrared illumination and its reflection patterns from the cornea of a subject to track and record the gaze locations on the screen.

### Procedure

Each participant solved approximately 10 six-letter anagrams during approximately a half-hour session. Anagrams were selected from a set that was repeated between participants. Participants were permitted to freely examine each puzzle and tried to solve it within 210 seconds.

The stimuli were presented on Tobii T60 with Tobii Studio software, which determined the location and timing of each fixation event on the screen with its proprietary fixation filter. We worked out the sequence of letters viewed for each anagram by a participant, by choosing the letter closest to a fixation location.

**Figure 1.**
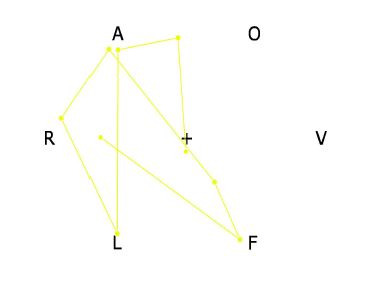
A sample stimulus with a 6-letter anagram overlaid with a partial gaze trajectory of a participant. The experiment started with the participant looking at the central fixation point (+ symbol), and the gaze moved to the top of the screen. Then, the participant looked directly at the letter A followed by L and R and so forth. The letter sequence inferred from this data would be ALRAFR. The fixation locations can be sometimes ambiguous, but moving letters farther apart and lowering the contrast improved the data quality.

**Figure 2.**
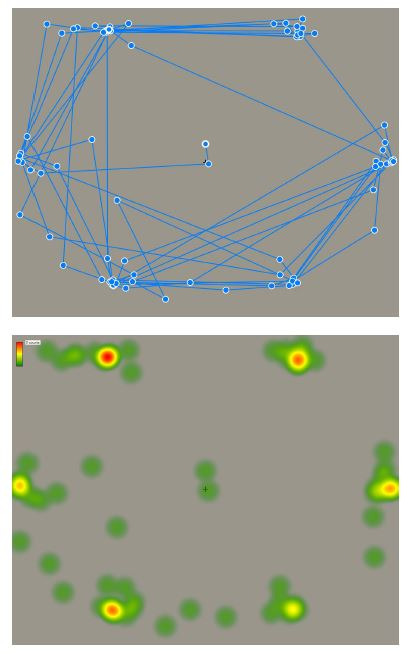
Reduced contrast and increased distance between the letters improved the data quality, by forcing the participants to look at the letters directly without using their peripheral vision. A sample gaze trajectory (Top) and the heat map of fixations (Bottom) are shown.

Pilot experiments revealed that participants sometimes did not look directly at the letters on the screen. If the letters were clearly visible as in Figure 1, the participants could see each letter without using foveal vision and hence without fixating directly on each letter. Reconstructing their gaze trajectories and inferring letter sequences was difficult. Therefore, we increased the distance between the letters and decreased the contrast and the font size in the stimulus image as in Figure 2, so that the participants would have to look at individual letters directly. These changes improved the quality of the data.

Statistical analyses were conducted using SPSS (IBM Corp., Armonk, NY) with alpha set to 0.05 for all analyses. The normality of data was assessed using Shapiro-Wilk tests, and outliers were assessed using Grubbs tests (13). Given the occurrence of non-normal distributions of several measures, we conducted nonparametric analyses on all data. Wilcoxon signed-rank tests were used for direct comparison of dependent samples, and Mann-Whitney U tests were applied to independent samples. Friedman tests were conducted to assess within-participant main effects, with Wilcoxon signed-rank tests conducted post hoc, when appropriate, with alpha adjusted using the Bonferroni correction method for multiple comparisons. Spearman’s rank correlation was used to assess monotonic relationships between variables. Confirmatory analyses conducted using parametric tests are not reported but yielded similar conclusions.

The pilot experiment revealed that subjects sometimes did not look directly at the letters on the screen. If the letters were clearly visible, as in Figure 1, the subjects could see each letter without having to use their foveal vision and hence without fixating directly on each letter. Reconstructing their gaze trajectories and inferring letter sequences would be difficult. Therefore, we increased the distance between the letters and decreased the contrast and the font size in the stimulus image, so that the subjects would have to look at individual letters directly. These changes improved the quality of the data.

## Results

### Result #1: Habitual scanning patterns strongly influence the gaze sequence

**Figure 3.**
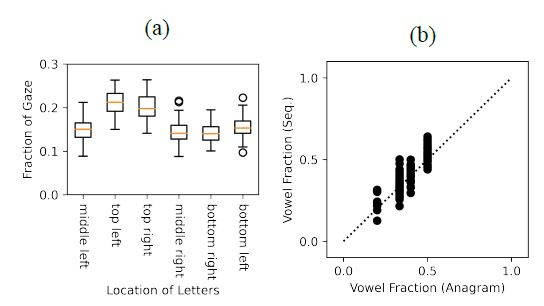
Single-letter gaze patterns. (a) Fraction of gaze time across six letter positions (b) Correlation between the fraction of vowels in presented anagrams and the fraction of vowels in participant letter sequences (N=29).

Participants looked at the top letters in the stimulus image most frequently (for example, the letters A and O in Figure 1), as shown in Figure 3(a). On average, each of the two letters placed at the top of a six-letter anagram was viewed approximately 20% during the trial, while the other four letters placed in the middle and bottom portions of the stimulus image were viewed 15% per letter. Friedman tests revealed significant main effects of letter position on cumulative fixation durations (N=86, χ2=228.37, p<0.001). Wilcoxon signed-rank tests conducted post hoc demonstrated that fixation durations for top left and top right positions did not differ (Z=1.78, p=0.076) but that participants fixated on these positions significantly more than all other positions (all p’s <0.001). No other pairwise comparisons reached statistical significance, with the exception that the fixation frequency for the bottom left position was greater than that for the bottom right (Z=4.79, p<0.001). This pattern of results is not surprising because people tend to look at a document, a screen, or a scene from top to bottom.

Whether a letter is a vowel or a consonant did not strongly influence the viewing frequency, as the fraction of vowels in the anagram correlates linearly with the fraction of vowels within the letter sequence (Spearman’s rho=0.87, N=113, p<0.001), as shown in Figure 3(b).

**Figure 4.**
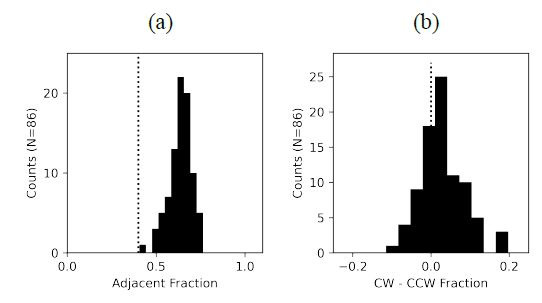
Sequential gaze patterns. (a) Fraction of adjacent letters viewed; (b) Difference in a fraction of clockwise (CW) versus counter-clockwise (CCW) gaze sequences

The following analysis examined the sequential gaze patterns. Would the participants look at the nearby, adjacent letters more often than the letters that are placed farther apart? For example, in Figure 1, AO and AR are adjacent, while AV and AE are non-adjacent pairs. We counted the number of times the next letter viewed by a participant was adjacent to the letter most recently viewed and compared it to the number of times the next letter was not adjacent. The fraction of times participants looked at an adjacent letter for each anagram trial was on average 0.64, as shown in Figure 4(a), indicating that more than 50% of the time, the participants looked at the letter either immediately to the left or right of the currently viewed letter. A Wilcoxon signed-rank test demonstrated that the distribution was significantly higher than the predicted value of 0.4 expected from a random distribution, given that 2/5 letters were adjacent (Z=9.17, p<0.001).

Furthermore, the participants tended to look at the adjacent letters in a clockwise direction more often than in a counterclockwise direction. In Figure 1, AO and OV are clockwise, while OA and AR are counterclockwise. The difference between the fraction of the clockwise scanning sequences and the counterclockwise sequences for each trial is, on average positive, indicating that clockwise viewing was more common, as shown in Figure 4(b). A Wilcoxon signed-rank test demonstrated that this distribution was significantly higher than zero (Z=3.72, p<0.001), the value predicted based on a random sequence of directions.

### Result #2: The knowledge of language statistics influences problem-solving behavior

We designed this experiment so that the solution to an anagram puzzle could not be found trivially by looking only through adjacent or clockwise letters. The participants had to explore different letter combinations that may not be conveniently (adjacently or clockwise) located relative to each other. For example, the solution to the anagram in Figure 1 involves combining letters far apart (LA) and arranged in a counterclockwise sequence (VO).

At the same time, a brute-force, exhaustive consideration of all possible combinations would be inefficient and unreasonable because the number of permutations increases rapidly with the length of letter combinations. For example, with six different letters, there are

30 (= 6x5) different unique 2-letter combinations (bigrams) to consider for a solution. Similarly, when a subject considers a combination of three letters (trigrams or 3-grams) within a six-letter anagram, there are 120 (= 6x5x4) possible combinations. Similarly, there are 360 4-grams, etc. The number of permutations would be even larger if we consider a permutation with replacement.

**Figure 5.**
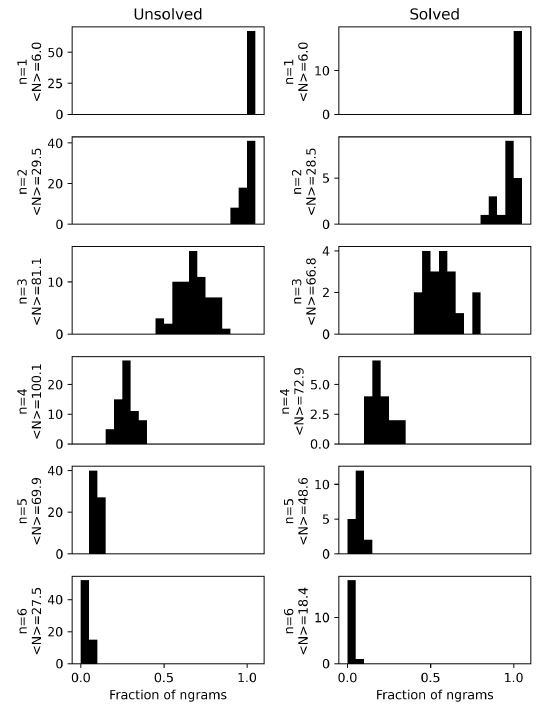
Consideration of n-grams (n = 1, 2, 3, 4, 5, 6) for 6-letter anagrams for (a) solved and (b) unsolved anagrams. For n = 2, there are 30 possible bigrams. For n = 3, there are 120 possible trigrams. The number of distinct n-grams within each letter sequence is counted and presented here as histograms.

When the fraction of unique n-grams viewed during each anagram trial is plotted as a histogram, the distribution shifts from 1.0 when n = 1 to lower fractions for large n, as shown in Figure 5. In other words, the participants looked at all individual letters (n = 1) and most of the possible bigrams (n = 2) during each trial, but they viewed only a small fraction of possible n-grams of higher lengths (n > 2). In other words, problem-solving does not involve a brute-force exploration of all possible solutions. Instead, a participant searches within a smaller space of hypotheses, presumably guided by prior knowledge of the English language.

Bigrams proved to be an appropriate length of letter combinations to analyze more deeply. Because there are too many possible combinations with more letters, the sample size for each n-gram with high n (n > 2) would be quite small. In contrast, the bigrams (n = 2) offered enough variety and enough appearances within each trial.

**Figure 6.**
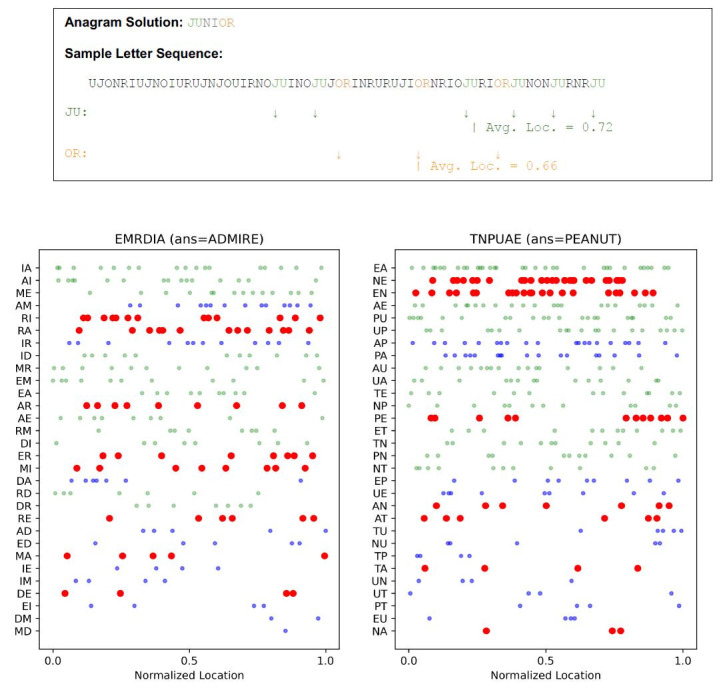
Sample letter sequences. Top: For a given letter sequence, we can locate the occurrences of a particular bigram (in this example, JU and OR) and determine its frequencies and average location. Bottom: The occurrences of different bigrams are shown as dots in each scatter plot. The horizontal axis represents the normalized length of the full letter sequence. The bigrams whose letters are adjacently located are shown in green. Non-adjacent, common bigrams in the English language are shown in red. The rest of the bigrams are shown in blue. There are 30 unique bigram combinations with six letters, and 12 bigrams are from adjacent letters. The example on the left is for an anagram EMRDIA, where these six letters were displayed clockwise. EM, MR, RD, DI, IA, AE, and their reverses (ME, RM, etc.) are adjacently located in this case.

To visualize and compare bigram appearances within a letter sequence, we normalized the length of each trial. Shown in Figure 6 are sample letter sequences. The top example shows a trial with an anagram whose solution was the word, JUNIOR. For this anagram, there are six letters and 30 possible bigrams (JU, UN, NI, etc.). The bigram JU is the first bigram of the solution word, and the bigram OR is the last bigram of the solution word. We can track the location of each bigram and calculate its frequency. In addition, we can also quantify their average location by using a normalized length scale where the full letter sequence has a length of 1.0. For example, if a certain letter or letter combination appeared at the beginning of the sequence, its position would be 0.0, and if it appeared in the middle, its position would be 0.5. An average location for a given letter combination can be calculated by combining these values.

Two fuller examples of anagram-solving patterns are presented in the bottom two panels in Figure 6. Each dot shows the location of a bigram within the letter sequence. 30 possible bigrams are listed in descending order of their frequencies. The green dots denote bigrams that are adjacently located in the anagram puzzle and are viewed very often, as expected from Figure 4(a). The red dots represent bigrams that satisfy two conditions: these bigrams are frequent in the English language and are not adjacently located in the puzzle. Some of these particular letter combinations were also viewed frequently, even without being adjacent. The rest of the bigrams are displayed as blue dots. These two examples illustrate the major trends observed in the experiments: the location of letters and the letter statistics of the language strongly influence the participants' gaze.

**Figure 7.**
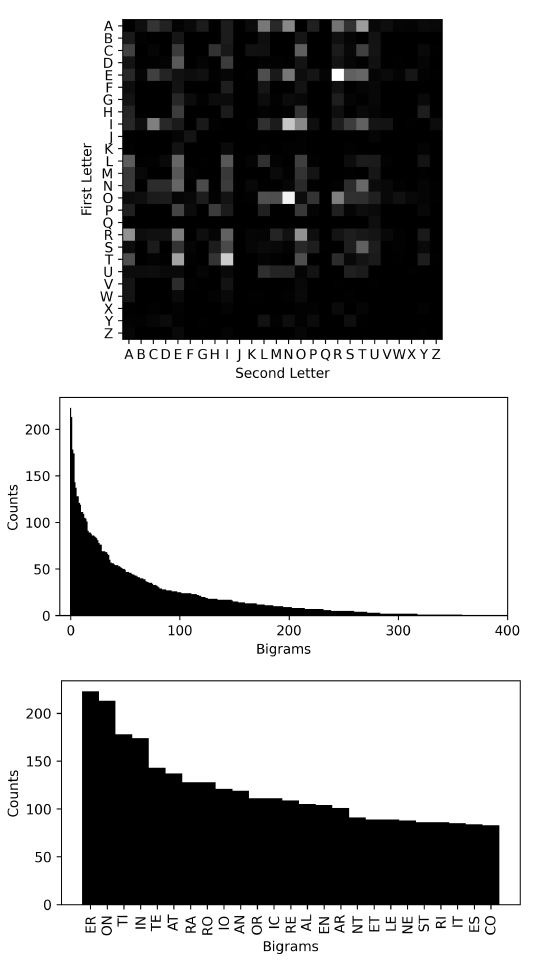
Bigram Statistics from Bourane and Ford (3). The top array shows the bigram statistics as a heat map, where the first letter in each bigram is displayed vertically and the second letter, horizontally. The following bar graph shows the ordered frequency of each bigram. The counts on the vertical axis are the number of occurrences per 10,000 bigrams without considering the position of the letter pairs within the word. The top 25 bigrams are shown at the bottom. The most frequent bigrams are ER, ON, TI, IN, TE, AT, RA, RO, IO, AN, OR, IC, RE, AL, EN, AR, NT, ET, LE, NE, ST, RI, IT, ES, CO, etc.

The letter statistics in the English language have been studied extensively (12). Bourane and Ford (3) examined the statistics of letters in English words and reported that the most frequent bigrams are: ER, ON, TI, IN, TE, AT, RA, RO, IO, AN, OR, IC, RE, AL, EN, AR, NT, ET, LE, NE, ST, RI, IT, ES, CO, etc. On the other hand, the least frequent ones include ZE, HT, BJ, XX, JH, etc. Their results are summarized in Figure 7.

All participants of our experiment were proficient in English and, therefore, would have developed instinctive familiarity with which letter combinations are more likely than others. A sensible approach for finding a solution to an anagram is to explore and consider letter combinations that are more frequent in English. However, as shown in Figures 3 and 4, the participants have a few habitual tendencies when scanning a stimulus image. Therefore, to compensate for the bias toward viewing adjacent letters, we analyzed only non-adjacent letter pairs and asked whether common bigrams (common according to the language statistics as in Figure 7) were viewed more frequently than rarer bigrams. This comparison was made by calculating the Z-scores for the most common and least common bigrams within each letter sequence. For example, in Figure 6, with an anagram EMRDIA, the count of bigram RI was subtracted by the average of other bigrams’ counts and was divided by the standard deviation of counts. According to Bourane and Ford (3), RI is one of the common bigrams in the English language. The bigram count for IA has not been used because the letters I and A were adjacent. A positive Z-score indicates that the frequency of this bigram is higher than the average, and a Z-score greater than 1 suggests that this count is more than one standard deviation above other bigram counts during the trial.

**Figure 8.**
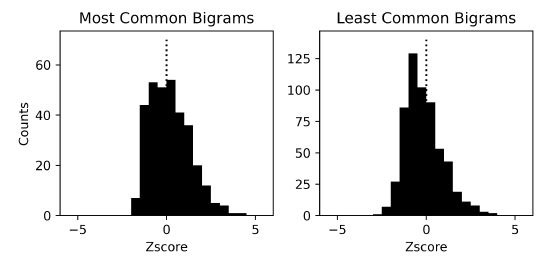
Histogram of Z-scores of non-adjacent bigram counts. The count of each bigram within a letter sequence was compared against the counts of other bigrams using a Z-score (mean subtraction followed by a division by the standard deviation). We analyzed letter sequences that were longer than 100 letters. The bigrams composed of adjacent letters or the bigrams that are parts of a solution word were not included in the analysis.

The Z-scores of the bigrams that are most and least common in the English language are presented as two histograms in Figure 8. The Z-scores of 50 most common bigrams were, on average positive, while the Z-scores of 500 least common bigrams were negative, with the mean Z-scores of 0.23 and -0.13, respectively. However, Wilcoxon signed-rank tests revealed that while the distribution of Z-scores of the least common bigrams differed from zero (Z=5.20, p<0.001), that of most common bigrams did not (Z=0.99, p=0.321). Thus, while participants viewed the more common bigrams to a similar extent to the average, they showed a markedly reduced likelihood of viewing the least common bigrams. Direct comparison of the two distributions of Z-scores using a Mann-Whitney U test demonstrated that participants viewed the least common bigrams less often than the most common bigrams (N=913, U=113,379.5, p<0.001), illustrating their utilization of linguistic knowledge as they search for the solution of an anagram puzzle.

We note that, in addition to removing adjacent bigrams in this analysis, the bigrams that make up a solution of the anagram were also excluded in this analysis, as the solution bigrams may affect the gaze patterns (rather weakly as discussed in the next section). Nevertheless, the major trend in Figure 8 remained the same regardless of the inclusion or exclusion of the solution bigrams. We also note that in calculating the Z-scores, the set of uncommon bi-grams was larger (500 least common versus 50 most common bigrams), because many uncommon bigrams (like ZZ or QQ) would not appear in the anagrams. We observed that the average Z-scores for most/least common bigrams were positive/negative, respectively, over a wide range of the bigram pools.

### Result #3: The gaze patterns on a suggestive solution bigram do not differ whether the participant was able to solve the anagram puzzle or not

An anagram eye-tracking study by Ellis et al. (8) included a distractor letter that was not part of a solution. Their study reported that approximately two seconds before reaching a solution, participants gradually began to dwell more on solution letters than distractor letters, even when participants reported that a solution suddenly emerged in their minds.

**Figure 9.**
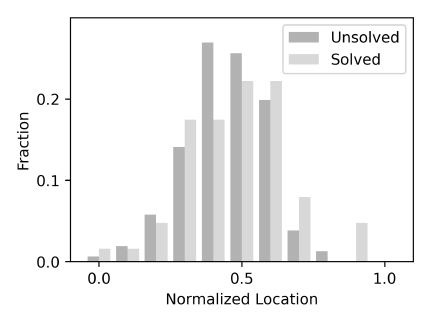
Average normalized locations of the first or the last solution bigrams. For the trials when the anagram puzzle was unsolved, the average location was 0.50 with an SEM of 0.011, and for the solved trials, the average location was 0.52 with a standard error of 0.022.

We explored whether a similar trend might be observed in our data by analyzing the average bigram locations of the first and the last bigrams in the solution word. These two bigrams that appear at the beginning and end of the solution word, of course, are highly suggestive. However, when a participant solved the anagram, the average location of these bigrams was only slightly later than the average location of unsolved trials, as shown in Figure 9. A Mann-Whitney U test revealed no significant difference in normalized location between solved and unsolved trials (N=219, U=4580, p=0.431). Thus, our analysis indicates that the gaze patterns between the solved and unsolved trials were more similar than different.

## Discussion

We examined problem-solving behavior using anagram puzzles. The gaze patterns of participants revealed that their prior habits and linguistic knowledge influence their gaze patterns. We showed that participants tended to view letters positioned at the top of the screen more frequently than those positioned in the middle or at the bottom. Further, participants were more likely to view adjacent letters than non-adjacent letters and tended to scan letters in a clockwise direction. The fraction of unique letter sequences (n-grams) viewed decreased as a function of n-gram length, suggesting that the problem-solving process did not involve a brute-force exploration of all possible solutions, and particular letter sequences viewed were influenced by letter sequence probabilities in the English language, as less common bigrams were viewed less often than more common sequences. Finally, gaze patterns of suggestive solution sequences occurred at similar average normalized locations in the gaze sequence of unsolved and correctly solved trials, suggesting that these suggestive bigrams were not viewed any more frequently toward the end of the solved trials.

Our data showing a bias in gaze position toward the top of the anagram (top left and top right) is reminiscent of position effects demonstrated in other visuomotor paradigms. For example, Durgin et al. (7) showed a prominent upper-left gaze bias in a visual search task, reflecting a habit of left-to-right directional scanning associated with reading (30). However, the finding that it has been observed in human infants and across several species, including non-human primates and dogs (14), as well as the finding that left bias can occur in the absence of explicit task demands (7), suggests that it may in part reflect a more general cognitive strategy possibly associated with cortical hemispheric dominance. The presence of top bias, as observed in the present study, has not been as extensively reported. However, Ryan et al. (24) demonstrated a top-to-bottom eye scanning bias in a choice task involving multi-attribute information.

Previous studies have utilized eye-tracking procedures to examine the impact of linguistic knowledge on problem solving. For example, Ellis and Reingold (9) showed that the central presentation of a three-letter word as a component of an anagram inhibited task performance relative to that on non-word trials, illustrative of the Einstellung effect, despite greater exploration of the remaining peripheral letters on word trials. Lapteva (21) further suggested that the frequency of the solution anagram word (in Russian) influenced both solution performance and solution strategy, in that less frequent words took longer to solve than more frequent words, and distractor letters were viewed less often during the solution of higher frequency words. The present finding that participants viewed less frequent bigrams less often than more frequent bigrams when solving anagrams extends these findings to suggest that implicit knowledge of letter sequence statistics is utilized in the problem-solving strategy. It would be helpful to explore this finding in the future using participants proficient in languages other than English, particularly in languages in which letter sequence probabilities differ from English. One might also predict that individuals proficient in English as a second language but who do not use it daily might be more influenced by letter statistics in their native language. This comparison might help confirm that knowledge of letter statistics, and not more general cognitive processes, are responsible for the observed pattern.

In contrast to our prediction, we found that gaze patterns of suggestive solution bigrams did not differ across the course of each trial for unsolved and correctly solved anagrams. We predicted that the bigrams consisting of the first two letters or the last two letters of the anagram solution are highly suggestive and, therefore, would be viewed more often later during correctly solved trials as participants explored various letter combinations and approached the correct answer. Our reasoning was based on the finding reported by Ellis et al. (8) that participants tended to dwell more on solution letters than distractor letters later in the sequence, a finding conceptually replicated by Lapteva (21). Although the present study did not utilize distractor letters, two results from the distractor studies are of interest related to the lack of effect we observed. First, an increase in viewing of suggestive solution bigrams may occur only during the last few seconds prior to the solution. If so, our focus on normalized location may not be sensitive enough to reveal an effect. We also note that there is a limitation on the generalization of results due to the small sample size of our study. It would be interesting to apply other metrics or approaches, such as entropy or ScanGraph (6), especially closer to the end of each trial. Second, as mentioned above, Lapteva suggested that the bias against viewing distractor letters was less likely to occur when the anagram solution was a lower-frequency word. This finding raises the possibility that solution word frequency may have influenced our results. It would be interesting to evaluate this possibility explicitly in a follow-up study involving high- and low-frequency solution words.

Using anagram puzzles for an eye-tracking experiment can elucidate a few fundamental issues in effective problem-solving: the tradeoff between exploration and exploitation as well as the inherent properties and traits of the problem-solving apparatus (e.g., biological brain versus computer). The participants in our study utilized a serial mode of problem-solving, where they tried out various letter combinations until a solution word was found. This approach is reminiscent of a hunter who follows a trail of prey until it is captured. It is a sensible approach for human participants. In contrast, consider an approach that may be implemented on a computer. An anagram-solving algorithm may start with a database of all six-letter words and filter out the words that do not have the letters in the anagram puzzle. Such a process of elimination would be efficient on a computer, but not for a human whose working memory is limited and error-prone. There has also been a report that humans do not consider subtractive solutions or strategies well (1).

There are also some challenges with anagram puzzles. For example, decoupling the effects of letter locations and letter statistics can be challenging. The human participants are susceptible to priming (31; 5; 18), so their solution-seeking patterns are influenced by their encounters and experiences before the experiment, and the solution from the prior trials may even influence the subsequent trial. The set of anagrams that are viable (i.e., familiar-enough words with a fixed number of letters) can be limiting. Nevertheless, it offers interesting avenues of research. Potential future works may employ anagrams with multiple solutions (e.g., DANGER and GARDEN), with a different letter or word statistics (e.g., anagrams chosen from statistically-distinct letter combinations), or with temporal variations (e.g., changing letters over time).

In conclusion, effectively incorporating prior knowledge and experiences can aid problem-solving. It will be intriguing to study whether and how people might shift the exploitation-exploration ratio (4), for example, as more information is gathered through multiple anagram rounds. That is, how do people gather the meta-level knowledge about the problem, and how does it affect their problem-solving behavior? How do people decide on the right balance between exploiting prior experiences and exploring new possibilities? Answering those questions is particularly important in education, because its goal is not just to impart specific domain knowledge, but to help the learners judiciously integrate their prior experiences within a new context while continuing to explore and acquire new knowledge and skills.

### Ethics and Conflict of Interest

The authors declare that the contents of the article are in agreement with the ethics described in http://biblio.unibe.ch/portale/elibrary/BOP/jemr/ethics.html and that there is no conflict of interest regarding the publication of this paper.

### Acknowledgments

This research was partly supported by Drew Summer Science Institute (DSSI). Adwik Rahematpura participated in the early phase of this project.
